# The skin microbiome of vertebrates

**DOI:** 10.1186/s40168-019-0694-6

**Published:** 2019-05-23

**Authors:** Ashley A. Ross, Aline Rodrigues Hoffmann, Josh D. Neufeld

**Affiliations:** 1 University of Waterloo, 200 University Avenue West, Waterloo, Ontario N2L 3G1 Canada; 2 Present address: Ontario Veterinary College, University of Guelph, 419 Gordon St, Guelph, Ontario N1G 2W1 Canada; 3 Department of Veterinary Pathobiology, College of Veterinary Medicine & Biomedical Sciences, Texas A&M University, 660 Raymond Stotzer Pkwy, College Station, TX USA

**Keywords:** Skin microbiome, 16S rRNA gene, Vertebrates, High-throughput sequencing, Mammals, Amphibians, Birds, Reptiles, Fish

## Abstract

The skin constitutes the primary physical barrier between vertebrates and their external environment. Characterization of skin microorganisms is essential for understanding how a host evolves in association with its microbial symbionts, modeling immune system development, diagnosing illnesses, and exploring the origins of potential zoonoses that affect humans. Although many studies have characterized the human microbiome with culture-independent techniques, far less is known about the skin microbiome of other mammals, amphibians, birds, fish, and reptiles. The aim of this review is to summarize studies that have leveraged high-throughput sequencing to better understand the skin microorganisms that associate with members of classes within the subphylum Vertebrata. Specifically, links will be explored between the skin microbiome and vertebrate characteristics, including geographic location, biological sex, animal interactions, diet, captivity, maternal transfer, and disease. Recent literature on parallel patterns between host evolutionary history and their skin microbial communities, or phylosymbiosis, will also be analyzed. These factors must be considered when designing future microbiome studies to ensure that the conclusions drawn from basic research translate into useful applications, such as probiotics and successful conservation strategies for endangered and threatened animals.

**Electronic supplementary material:**

The online version of this article (10.1186/s40168-019-0694-6) contains supplementary material, which is available to authorized users.

## Introduction

Skin microbiome research seeks to better understand the largest organ of the body by providing information on the processes by which a host organism evolves in association with its diverse collection of fungi, bacteria, archaea, and viruses [1], characterizing the immune system and diagnosing illnesses [2, 3], and exploring the etiologies of diseases [4–6]. The advent of high-throughput sequencing has greatly expanded knowledge of the skin microbiome and its implications for health. For example, it is now recognized that humans are uniquely colonized by skin microbial communities that are linked to diet [7], age [8, 9], and the specific body region sampled [10, 11]. These baseline data are important for understanding how skin microbiota contribute to skin health and disease.

**Fig. 1:**
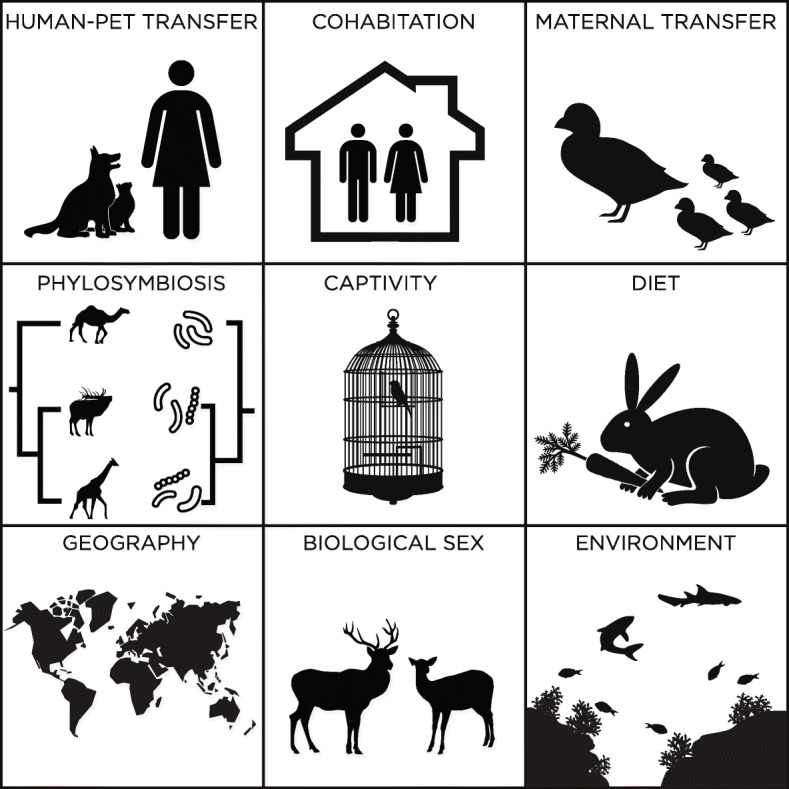
Examples of factors that influence the vertebrate skin microbiome

The majority of skin microbiome studies have focussed on humans, companion and domestic animals, and amphibians. Fish and birds have received substantially less attention, and many existing studies are cultivation-based. Few studies have explored the skin microbiome of reptiles [1]. The aim of this review is to summarize studies leveraging high-throughput sequencing to better understand the skin microorganisms that associate with members of classes within the subphylum Vertebrata (Additional file 1: Table S1). Specifically, links will be explored between the skin microbiome and vertebrate characteristics, including geographic location, biological sex, diet, captivity, maternal transfer, and phylosymbiosis (Fig. 1). This review will mainly focus on non-human animals because other reviews summarize human skin microbiome research [12–15].

### Vertebrate skin physiology

The outermost layer of mammalian skin is the epidermis, which is frequently studied in part due to its non-invasive sampling protocols and direct contact with the surrounding environment. It is here that commensal microbiota protect the body from transient microorganisms [12] with the potential to cause disease by either producing inhibitory compounds [16] or competing for resources [17]. The epidermis is constantly shedding and it is considered to be a hostile environment, compared to the gut or mouth, because of its lower temperature, pH, and moisture levels, coupled with relatively high salt and antimicrobial concentrations [18]. Estimates suggest that between 10^6^ and 10^9^ microorganisms/cm^2^ are present on human skin [19, 20]. This difference of several orders of magnitude can be attributed to sampling different body locations. Although more invasive techniques, such as skin biopsies or scrapes using a surgical scalpel blade, collect a higher number of microorganisms than superficial skin swabs, there are no consistent depth-dependent differences in detected microbial communities [20]. A recent study comparing swabbing and tape-stripping techniques observed no differences between these techniques [21].

The Class Mammalia includes the closest evolutionary relatives of humans. Non-human mammals typically possess denser fur over a larger proportion of their bodies. Sebaceous glands and two types of sweat glands are present on mammals, including apocrine and eccrine, that may each select for distinct microbiota. Sebaceous glands produce oily viscous exudates. The large and spongy apocrine glands are associated with fur and hair [22]. In contrast, the eccrine glands, which are small and associated with pores, are predominant in human and non-human primate skin [22]. Other mammals have a wide distribution of apocrine glands throughout their bodies, with eccrine glands only on feet.

The skin of avian reptiles (Class Aves, herein referred to as “birds” for clarity) has distinct physiological features from mammals. Although the most striking difference between birds and mammals is the presence of plumage, birds also have a thinner epidermis, no sebaceous glands, and a higher proportion of lipids in the transitional layer of the epidermis [23]. Birds are closer relatives of reptiles, especially modern-day crocodiles, than mammals. Their feathers are considered modified scales and a component of the integument, which is the outer protective organ system that includes the layers of skin, glands, hair, and nails in vertebrates [24]. Moreover, birds possess avian scales on their feet and only a single gland type [25]. The uropygial glands (“preen glands”), which are located in the dorsal region of most birds, exude an oily secretion that is used to coat feathers.

Non-avian reptiles (Class Reptilia, herein referred to as “reptiles” for clarity) include crocodiles, turtles, snakes, and lizards. This class of amniotes (“membrane surrounding the fetus”) represent the first animals to transition to land, which resulted in accompanying shifts to their integument. Reptiles were also the first organisms to evolve a stratum corneum (i.e., horny outer skin layer) with multiple layers and programmed cell death, coupled with additional lipids to prevent water loss on land [26]. A terrestrial lifestyle also led to the loss of gas exchange and mucous, which occurred approximately 340 million years ago [26]. The pleated-sheet beta-keratin polypeptides involved in creating sauropsid feathers, scales, and claws are distinct from the helical alpha-keratin polypeptides that form hair [25].

Amphibians, such as frogs and salamanders, possess a thin and persistently moist layer of skin that is water permeable and able to undergo gas exchange [27]. Unlike the other vertebrate classes, their skin contributes to respiration and osmoregulation while functioning as an innate immune organ [28]. Amphibian skin anatomy has been expertly reviewed elsewhere [28]. In brief, these tetrapods were the first vertebrates to evolve corneous cells [26], which form a protective external envelope around the organism and aid in terrestrial survival. An absence of protective integument layers, namely feathers or fur, makes them particularly susceptible to skin diseases [6]. Additionally, their skin is covered in a sugar-rich mucosal layer that can serve as a growth substrate for pathogenic bacteria and fungi. Consequently, many amphibian microbiome studies have focused on elucidating the differences between infected and uninfected animals in an attempt to create conservation strategies to prevent species extinctions. In particular, studies have focused on chytrid fungus, which was recently declared to have caused the most devastating recorded loss of biodiversity that can be attributed to disease [29]. As a result, the amphibian skin microbiome is better characterized than several of the other vertebrate classes.

Fish represent an evolutionarily diverse clade of six vertebrate classes. Their scales are formed in the mesoderm layer and they do not possess keratin and a corneous cell envelope, in contrast to keratinized reptilian scales that are formed in the epidermis [30]. Like amphibians, fish possess a layer of mucous that surrounds the epidermis [31] and represents an additional critical barrier between the animal and its aquatic environment. The mucous is a complex viscous mixture of immunogenic compounds, such as mucins, immunoglobins, lysozyme, antimicrobial peptides, and defensins that contribute to both innate and adaptive immunity [2, 32]. Despite these bactericidal compounds, the mucous layer also possesses numerous sugars and amino acids suitable for bacterial growth [33].

## Microbial diversity and composition of vertebrate skin

### Non-human terrestrial mammals

Despite the importance of the mammalian microbiota, only a few skin microbiome studies have been conducted on non-human mammals (key papers included in Additional file 1: Table S1). Initial culture-based studies of dogs and cats reported minimal skin bacterial diversity [34]. Other studies showed that squirrels, raccoons, cattle, pigs, sheep, and dogs were dominated by *Micrococcus* and *Staphylococcus* [35], with *Staphylococcus* being detected in 100% of pigs and cows, 90% of humans and horses, 77% of laboratory mice, and 40% of dogs [36].

Similar to human skin microbiome studies, the ability to use high-throughput sequencing has expanded our understanding of vertebrate skin microbial diversity. A large study using superficial skin swabs to evaluate the skin microbiome of wild, farm, zoo, and household animals found the majority of animals had higher diversity and distinct skin microbial communities, as compared to human samples [37]. The study evaluated skin samples from the back, torso, and inner thigh regions and found no significant variation among hair-covered body locations. The differences between human and animal skin were largely driven by a decreased relative abundance of *Actinobacteria* on mammalian skin, with corresponding increases in the abundances of *Chloroflexi* and *Bacteroidetes*. A study that compared human and primate axillae also found that human skin communities were unique compared to non-human primates, including gorillas, chimpanzees, rhesus macaques, and baboons [38]. Other 16S rRNA gene studies of healthy and allergic dogs [39] and cats [34, 40] also observed higher species richness and diversity on skin from animals compared to human studies, with relatively higher abundances in bacteria in the phyla *Proteobacteria* and *Bacteroidetes*. Mucosal surfaces of companion animals were inhabited by less diverse bacterial communities compared to haired skin. However, significant variations in community structure have also been observed among different haired anatomic regions in horses [41], suggesting that additional factors influence microbial communities on an animal, such as contact with other vertebrates and the environment.

Eukaryotic microscopic fungi are an additional group of microorganisms within the skin microbiome (i.e., the “mycobiome”). Fungi are less abundant than bacteria according to human skin metagenomic analysis [42]. Dogs and cats [43, 44] are also colonized by diverse fungal communities, which often vary across haired and mucosal surfaces and disease status. Their mycobiome seems to be more diverse than the human mycobiome and dominated by fungal genera including *Cladosporium*, *Alternaria*, and *Epicoccum*, whereas the genus *Malassezia* was recorded at > 90% relative abundance in human skin, except the feet [45].

Host species is an important predictor of skin microbial communities. Indeed, a survey of 38 mammalian species determined that host order and species were the most significant influences on skin microbial communities [37]. This has been also observed in North American bats [46] and early culture-based studies of mammalian skin determined that non-human animals had distinct dominant staphylococci from humans [36]. Even breed can influence the skin microbiome of mammals, as has been recently demonstrated cats [44].

In humans, overall microbial community composition is influenced by biological sex [47], age [8, 48], diet [7, 49, 50], use of hygiene products [10], ethnicity [51], cohabitation [52], habitats, and geographic location [53, 54] (Fig. 1). In terms of biological sex, only a few studies have identified significant differences between male and female mammals, including captive red kangaroos in Canada [37] and wild bank voles in Ukraine [55]. Although the link between diet and skin microbiota has not been established, diet has been linked to the composition of the gut microbiome in healthy mammals [56], including carnivores, omnivores, and herbivores. Thus, diet is presumed to also impact the skin microbiome and influence skin diseases. Canine odor is another factor associated with changes in microbial communities, with bloodhound dogs with malodor having lower diversity than controls, mainly due to higher abundances of the genera *Psychrobacter* and *Pseudomonas* [57].

Similar to humans, cutaneous microorganisms are transferred maternally to non-human vertebrates (Fig. 1). The pouch of the Tasmanian devil (*Sarcophilus harrisii*), where the marsupial protects its developing offspring, is associated with similar microbial community composition to its skin, in terms of phylotype richness and the number of operational taxonomic units (OTUs) present. Skin samples were clustered with pouch samples instead of mouth and gut samples [58]. Significant differences were also observed among these specimens, with an increase in *Clostridia* and decrease in *Bacilli* for pouch samples.

Geographic location is an important factor influencing the skin microbiome of mammals (Fig. 1). Two studies of North American bats concluded that geographic location was an important predictor of microbial community composition [46, 59]. Within Southwestern Ontario (Canada), the source location for sampled mammals was a significant factor influencing skin microbial communities, albeit exhibiting less influence than host taxonomic order [37]. A recent study identified that the skin microbiome of humans and three species of pigs differed among inhabitants from high and low altitudes [60]. In particular, *Arthrobacter*, *Carnobacterium*, *Cellulomonadaceae*, *Paenibacillus*, and *Xanthomonadaceae* were five taxa significantly increased in individuals from high altitudes [60]. Wild bank voles in Ukraine had shifts in their skin communities over large spatial distances between Kyiv and the Chernobyl Exclusion Zone, irrespective of skin radionuclide contamination [55]. One study also described seasonality to have an effect on the skin microbiota of dogs [61]; however, sampling across breeds was performed in a single year. The same study described that cohabiting individuals shared their microbiota, as previously demonstrated in humans [52].

Evidence suggests that companion animals and their owners transfer microorganisms to each other, and in turn, impact the detected human skin microbiome [62] (Fig. 1). Such evidence demonstrates that shedding of the skin microbiome impacts both the microbial community composition of inanimate objects and living macroorganisms alike. Indeed, exclusively indoor cats were shown to have similar microbial communities to their owners, compared to outdoor barn cats [37]. Animals that inhabit the same enclosed habitat, such as humans and their pets in a house [63], companion animals in a barn, or zoo animals in a cage, likely alter each other’s respective microbiomes. Built environment studies demonstrate that household surfaces are rapidly colonized with the microbiome of their inhabitants [64]. Therefore, the transfer of skin microbiota between animals can occur from either direct skin-to-skin contact or via indirect contact with shared surfaces. Although the direction of transfer can be difficult to determine in uncontrolled and complex environments, these transmission routes have important implications for the spread of infectious and zoonotic diseases. In turn, the environment is also likely an important source for new microorganisms to inhabit the skin, which can occur from direct contact with water, soil, or household surfaces. Within non-human mammals, Tasmanian devils had significant differences in skin microbial communities between wild and captive specimens, although larger differences were observed between gut microbiota [58]. Captive devils had elevated levels of *Mycobacterium*, a common cause of skin infections in captive facilities. Indoor and outdoor environments can also affect the skin microbiome of companion animals. Although cats primarily kept outdoors did not present higher microbial diversity as compared to indoor cats, their microbial structure and composition varied across these animals, with *Corynebacterium* spp., common bacteria on human skin, being more common on indoor cats [44].

Dysbiosis, defined as a shift from a normal microbiome, is associated with numerous skin diseases [4] and has been reviewed elsewhere for humans and domestic animals [65]. These polymicrobial diseases are complex and can involve the interactions of numerous microorganisms. Many pathogenic microorganisms compete directly for physical space and sources of food on human skin including sugars, ammonia, and amino acids [66], but possess virulence factors that harm the host in comparison to commensals. Commensal skin bacteria, such as *Staphylococcus epidermidis*, produce antimicrobial compounds to limit transient microorganisms from colonizing and appropriating resources. However, pathogens with pathogenicity islands are capable of outcompeting abundant commensals for resources [66], evading the host immune system, and subsequently lowering the abundance of typical healthy skin populations. Moreover, defects in the skin barrier may lead to penetration of pathogenic microorganisms and subsequent cutaneous inflammation, as has been shown in patients with atopic dermatitis [67–69]. Filaggrin is an important component of the skin barrier. Mutations in its encoding FLG gene results in a thickened, dehydrated stratum corneum and more clinically severe signs of disease [68]. Defects in the lipid bilayer, tight junctions, and proteases have also been associated with increased atopic dermatitis severity [69].

Microbial communities are typically more diverse on healthy skin, and there is evidence that microbial community composition affects several skin conditions in companion animals with atopic dermatitis and allergic skin diseases [39, 40, 43, 70, 71], bovine digital dermatitis [72], demodectic mange [73], white nose syndrome in bats [74, 75], and camel dermatophilosis [76] in other vertebrates. Dogs with skin allergies and atopic dermatitis exhibit lower bacterial richness [39] and diversity on their skin than their healthy counterparts [70], due to increases in proportions of *Staphylococcus pseudintermedius*. Although changes in diversity have not been observed in allergic cats, their skin is also inhabited by higher proportions of *Staphylococcus* spp. [40]. Horses have a stable skin microbiome that is able to return to its initial composition once a wound has healed [41]. During an experimentally induced wound experiment, the abundance of *Fusobacteria* and *Actinobacillus* increased during the early stages after wound formation. Unbandaged wounds had greater microbial diversity. This study recorded key information on temporal changes to the mammalian skin community throughout an ~80 day wound healing process and provided data that may inform veterinary practices for successful treatment of wounds. The aforementioned studies focused on reporting diversity as a proxy for health. These comparisons are based upon the community ecology perspective that diverse communities are more stable and resilient to external disturbances. Bioreactor experiments demonstrate that dynamically shifting communities can still be capable of maintaining stable ecosystem functions [77]. Subsequent human microbiome research shows that the healthy human skin microbiome is relatively stable over time due to fixed abundant species [78] and that subsequent decreases in diversity can result in disease. Atopic dermatitis treatments that increase microbial diversity ameliorate the condition [79] and provide a prime example of why researchers should continue to test for diversity when studying dysbiosis. Diversity should ideally not just be reported but should be further explored to determine how skin ecosystem diversity influences disease severity and response to treatment.

Digital dermatitis affects the hooves of cattle and results in lameness, corresponding to major economic losses to the agricultural industry [80]. Animals with digital dermatitis have higher bacterial diversity and increased prevalence of bacteria affiliated with *Bacteroidetes*, *Proteobacteria*, and *Spirochaetes*. In particular, *Treponema* spp. [81] are abundant in deep lesions and likely originate from the gut reservoir [72]. Sheep footrot is a similar infectious disease that results in lameness for entire sheep herds [82]. *Dichelobacter nodosus* likely initiates the disease, whereas *Fusobacterium necrophorum* plays a secondary role in infection [82]. Both digital dermatitis and sheep footrot are examples of polymicrobial diseases, where shifts in several skin microbiome taxa precede the onset of clinical symptoms. Dysbiosis also affects the fungal microbiota of vertebrate skin, with allergic dogs having lower diversity [83]. For dogs and cats with allergic skin disease, their mycobiota became very similar across different body sites [43]. Lastly, white nose syndrome devastates bat populations and is caused by the fungal pathogen *Geomyces destructans* [75].

### Aquatic mammals

The skin of aquatic mammals has been studied to further marine conservation efforts. To date, cetaceans such as humpback whales, dolphins, and killer whales have been sampled. Significant differences were found between the microbial communities of bottledose dolphins and killer whales [84]. Skin biopsies and sloughed skin from 56 humpback whales (*Megaptera novaeangliae*) from the North Pacific, South Pacific, and North Atlantic oceans demonstrated *Psychrobacter* and *Tenacibaculum* as the core genera present on these free-swimming whales [85]. The abundance of these two genera varied significantly between humpback whales undergoing anabolic and catabolic metabolic states [85].

The cetacean skin microbiome varies geographically [86–88] (Fig. 1). Specifically, offshore bottlenose dolphins have higher skin microbial diversity than their coastal counterparts, who were more similar due to exposure from coastal runoff [87]. The skin microbiota of humpback whales was distinct from the surrounding seawater [86]. Likewise, skin samples of captive dolphins are also associated with distinct microbiota according to the environment where they are kept, being significantly influenced by food and air. Each environment maintained a distinct microbiota despite exposure incidents, implying that few exposures lead to permanent colonization [89]. Future studies aiming to provide evidence to improve the conservation status of wild animals affected by skin diseases should therefore include sampling of wild animals for the most accurate skin microbial community information.

### Avians

Avian skin microbiota can be influenced by sex [90, 91], species [92], age, and habitat [90] (Fig. 1). European starlings (*Sturnus vulgaris*) and bluebirds (*Sialia sialis*) have distinct sex-dependent diversity associated with sampled plumage [90, 91]. These variations may be attributed to physiological variations between the sexes, such as pH [93]. In contrast, no differences were identified in the skin microbiota among male and female vultures [61]. The avian skin microbiota has also been linked to both nest location and age [90].

Birds are social animals whose sexual and social constructs aid in bacterial transmission [5]. For instance, the feathers from caged zebra finches (*Taeniopygia guttata*) infected with *Bacillus licheniformis* resulted in an oral-fecal-genital route of transmission. Preening led to autoinfection, which progressed to a sexual infection whose transmission rates varied by biological sex [5]. European starlings with larger brood sizes have more bacteria on their feathers [90]. Manipulating their brood size resulted in significantly different bacterial community composition on plumage, but not richness or feather degradation. Additionally, bluebirds sharing the same nest transmit plumage bacteria, based on results from culturing techniques [91]. Certain subsets of the microbiome are classified as “feather-degrading bacteria” and influence the condition of feathers and by extension avian health. These polyphyletic bacteria include OTUs affiliated with *Bacillus* spp. and hydrolyze ß-keratin [95], which is the predominant protein in feathers. The finding that nest sharing results in microbiome transmission has implications for the distribution of feather-degrading bacteria that are associated with body condition and feather coloration [91]. Recently, comparisons were made between three finch species [92]. Although these finches received the same diet and environmental exposures, each species had distinct overall skin communities, despite sharing conserved core OTUs. The authors hypothesized that the observed differences may contribute to odor production.

The eating habits of scavenger birds can alter the diversity of their skin microbiota. The skin microbiota of two species of New World vultures (*Coragyps atratus* and *Cathartes aura*) exceeds the diversity of their gut microbiota [94] (528 vs 72 OTUs, respectively). Frequent contact with carcasses may explain this increase in skin microbial diversity. *Clostridia* and *Fusobacteria* were dominant OTUs on vulture skin.

Very few virome studies involving the collection of nucleic acid sequences from the viral community in a habitat have been performed with animals. A single high-throughput sequencing study examined 15 healthy chickens (*Gallus gallus domesticus*) and determined that their skin was predominately inhabited by herpesvirus from the *Mardivirus* group [96]. The authors hypothesized that the viruses arose from vaccination or an asymptomatic infection. In addition, the skin virome of chickens differs from those of reptiles [97] and humans [42, 98]. Notably, chicken skin was absent of papillomaviruses and polyomaviruses that are typically detected on human skin [42, 98].

### Reptiles

Despite links to numerous skin infections caused by viruses, bacteria, fungi, and parasites, very few studies have focused on the reptilian skin microbiome. A study on the oral and skin microbiome of komodo dragons (*Varanus komodoensis*) elucidated that captive dragons and their enclosure had similar microbial community composition and species richness [99] (Fig. 1). The Komodo dragon skin microbiome had higher diversity than either the oral or fecal microbiome. Dominant skin phyla included *Bacteroidetes* and *Firmicutes*, which were present in equal proportions [99].

Several studies have focused on microorganisms that are causative agents of reptile skin diseases. Reptiles are prone to infection by a variety of predominately Gram-negative commensal bacteria, including *Aeromonas*, *Klebsiella*, *Proteus*, *Pseudomonas*, and *Salmonella* [100]. Fungal dermatitis in the USA has affected numerous reptilian species, including dusky pigmy rattlesnakes (*Sistrurus miliarius barbouri*), garter snakes (*Thamnophis sirtalis*), and ribbon snakes (*Thamnophis sauritis*) [101]. The fungus *Ophidiomyces ophiodiicola* currently causes high mortality in snakes across Europe and North America [102]. A study of Eastern Massasauga snakes (*Sistrurus catenatus*) determined that infected snakes were more likely to have high populations of *Serratia* and *Janthinobacterium*. In contrast, *Janthinobacterium* has been associated beneficially with salamander populations to prevent *Batrachochytrium dendrobatidis* infections [103], whereas *Serratia* has been observed in the skin microbiome of immunocompromised human patients [104]. Moreover, a subset of OTUs such as *Xylanimicrobium* and *Cellulosimicrobium* were reduced in infected snakes, further indicating that snake fungal disease shifts the skin microbiome [102]. Another study determined that microbial communities did not differ significantly between snake populations of timber rattlesnakes (*Crotalus horridus*) and black racers (*Coluber constrictor*), indicating that snake fungal disease studies on model organisms may widely apply to multiple snake species [105]. Future studies will be able to leverage these findings to investigate whether a “protective microbiome” may help conservation efforts. For example, it may be possible to create a skin probiotic culture from microorganisms that have been experimentally determined to be protective against skin diseases. Developing a stable topical treatment may prove useful to shift the microbiome, just as this strategy has been moderately successful with human gut probiotics [106].

Additional studies have focused on the reptilian skin virome in relation to disease. A skin microbiome study of reptiles focused on the lizard virome [97]. Multiple viruses were associated with lethal skin lesions, including *Ranavirus*, *Adenovirus*, and *Reovirus*. Herpesvirus is currently infecting both wild and captive turtles and tortoises resulting in necrotizing lesions [100]. Examples of affected species include Argentine tortoises (*Chelonoidis chilensis*), Mediterranean tortoises (genus *Testudo*), Pacific pond turtles (*Actinemys marmorata*), and painted turtles (*Chrysemys picta*). Fibropapillomatosis affects wild populations of marine turtles, especially the green, loggerhead, and olive ridley sea turtles (*Chelonia mydas*, *Caretta caretta*, and *Lepidochelys olivacea*, respectively) [100]. This viral infection has spread globally and there is no current protocol to prevent transmission within wild populations.

Other skin-associated infections, such as inclusion body disease (IBD), caused by a *Retroviridae* virus [107], are prevalent on multiple continents [100]. The distribution of IBD has primarily been reported on boid snakes, including Burmese pythons (*Python bivittatus*) and Boa constrictors (*Boa constrictor*) in Africa, Australia, Europe, and North America. Reptilian skin has been shown to harbor several viruses that lead to lesions and premature death [97]. Baseline high-throughput sequencing data of healthy and diseased skin states are required to implement conservation measures.

### Amphibians

Many amphibian species have had their skin microbiota sampled to establish a microbial baseline (Additional file 1: Table S1), which is particularly important because of declining amphibian populations due to skin fungal infections [108]. Wild tiger salamanders (*Ambystoma tigrinum*), western chorus frogs (*Pseudacris triseriata*), and northern leopard frogs (*Lithobates pipiens*) harbor a similar level of diversity as human skin [109]. Of the 18 bacterial phyla observed on amphibian skin, *Acidobacteria*, *Actinobacteria*, *Bacteroidetes*, *Cyanobacteria*, *Firmicutes*, and *Proteobacteria* were most abundant [109]. Amphibian host species was the most important predictor of community composition in a study of five species that included toads, frogs, and newts [110]. This trend in amphibians is further supported by a study of Panamanian frog species, which determined that there were key differences among hosts at bacterial taxonomic levels below the phylum level [111]. Red-backed salamanders (*Plethodon cinereus*) had eight core OTUs, including *Pseudomonas* [112], present on > 90% of specimens. Italian steam frogs (*Rana italica*) were characterized by 16 distinct phyla [113] with a fifth of all detected OTUs present in all subjects [113]. A culture-based study of Cascade frogs (*Rana cascadae*) enumerated 20 higher order taxa and 31 genera [114].

Transmission of skin bacteria to four-toed salamander (*Hemidactylium scutatum*) embryos has been observed [115]. These salamanders can use communal nests with eggs from a minimum of two females, which leads to higher rates of offspring survival. These communal nests were more likely to contain skin bacteria that inhibit the fungus *Mariannaea*, which is lethal to four-toed salamanders. Only 27% of females have these beneficial skin bacteria and having multiple females in contact with a nest resulted in higher survivability rates than solitary nests with lower amounts of antifungal bacteria in their skin communities [115].

Amphibians have different skin microbial communities depending on the current stage in their life cycle. Tadpoles are associated with distinct skin microorganisms before they undergo metamorphosis [110]. The common coqui (*Eleutherodactylus coqui*) has skin microbial communities that differ between juvenile and adult forms [116]. Female four-toed salamander populations within a single nest location shared a greater proportion of common bacteria than those detected as being shared among all sampled individuals from all nests [117].

Differences exist among the body regions of fire-bellied toads (*Bombina orientalis*), such that the dorsal sides of wild toads associates with higher diversity and richness than ventral sides, whereas captive toads exhibit the opposite result [108]. Because some non-human vertebrate studies used a single swab to sample all body locations, future skin microbiome research should sample multiple body locations per animal to assess skin community heterogeneity. Lack of sex documentation is especially prevalent for amphibian studies (Additional file 1: Table S1), due to the difficulty of non-invasive sexing methods.

Skin bacterial communities in amphibians are influenced by diet, and their microbiota may also influence their behavior (Fig. 1). For instance, providing captive red-eyed tree frogs (*Agalychnis callidryas*) with a carotenoid-rich diet increased skin bacterial richness and abundance, including increases in *Staphylococcus*, *Flavobacterium*, *Klebsiella*, and *Citrobacter* [118]. Odor cues produced by bacteria are involved in influencing the behavioral activities of the red-eyed tree frogs, including mating, marking territory, and recognition [119]. Determining the distribution of microorganisms on vertebrate skin has the potential to answer several questions about animal behavior that were raised previously [120], such as how animals recognize individuals and kin and assess mate quality and social relationships.

Geographic location and seasonal variability have both been associated with shifts in amphibian skin populations (Fig. 1). A large study on five different amphibian species (i.e., *Anaxyrus boreas*, *Pseudacris regilla*, *Taricha torosa*, *Lithobates catesbeianus*, and *Rana cascadae*) in the USA determined that wetland site was the largest predictor of skin microbial community composition within each species [110]. Variations in microbial communities based on the geographic location the host inhabits can be partially explained by the microorganisms collected from local abiotic environments. A study of red-cheeked salamanders (*Plethodon jordani*) demonstrated that sampled salamanders shared their most abundant bacterial taxa with the moist forest floor debris [121]. In contrast, skin swab samples of redback salamanders, eastern newts (*Notophthalamus viridescens*), and larval bullfrogs (*Rana catesbieana*) were distinct from the water they inhabited [122]; amphibians cohabitating the same pond was not a significant factor influencing their community structure [109]. Seasonal variation has been observed in the lowland leopard frog (*Lithobate yavapaiensis*), which may be linked to disease incidence because frogs are at increased risk of *B. dendrobatidis* infection during winter [116]. It has been hypothesized that reduced immunity caused by exposure to lower temperatures [116] and humidity [111] contributed to the temporal bacterial community changes.

Amphibian skin microbial communities may also be affected by contaminants in the surrounding environment, potentially reducing skin defenses and immunity [28]. A study of the Perez’s frog (*Pelophylax perezi*) demonstrated that frogs living in a metal-rich environment had distinct skin microbiome profiles from frogs in uncontaminated environments [123]. All frog skin samples revealed bacteria predominately from the *Actinobacteria* and *Alphaproteobacteria* taxonomic groups, whereas those from contaminated sites had more OTUs associated with acid-metal contaminated water, such as *Moraxella*, *Mycobacterium*, and *Hydrotalea*. Testing the surrounding soil or water for both biotic and abiotic composition may therefore add more insight into factors that influence skin microbial community composition.

Similar to data previously shown for mammalians [58], wild amphibians have higher bacterial diversity levels on their skin than the same species in captivity (Fig. 1). Wild red-eyed tree frogs (*Agalychnis callidryas*) had over twice the number of bacterial OTUs on their skin as their captive counterparts, demonstrating that captive animals have a significant decrease in diversity [118]. The Panamanian golden frog (*Atelopus zeteki*) shares approximately 70% of bacterial OTUs on their skin between wild and captive specimens, although significant differences in richness, community structure, and phylogenies still existed [124]. Overall, wild fire-bellied toads had higher diversity than captive toads, which varied based on the presence of *B*. *dendrobatidis* infection [108].

Approximately 30% of all amphibian species are threatened with extinction [125]. Given their sensitivity to skin infection, amphibian skin has been relatively well studied among vertebrate classes in an effort to prevent infections within wild populations, such as those linked to *Ranavirus*, mycotic dermatitis, and chytridiomycosis [16, 112, 126]. A variety of fungi have been cultivated from the skin of injured hellbender salamanders (*Cryptobranchus alleganiensis bishopi*), including *Acremonium*, *Cladosporium*, *Curvularia*, *Fusarium*, *Streptomycetes*, and *Penicillium* [127]. In this same study, isolated opportunistic bacterial pathogens included *Aerococcus viridans*, *Aeromonas hydrophila*, *Gordonai terrae*, *Granulicetella adiacens*, *Stenotrophomonas maltophilia*, and *Streptococcus pneumoniae*. The cutaneous microbiota of two giant salamander subspecies (*Cryptobranchus alleganiensis*) were studied to better understand why the Ozark hellbender subspecies is affected by chronic wounds, whereas the eastern subspecies is not [128]. Salamanders with wounds had higher OTU abundances than those without wounds, potentially indicating that commensal environmental and skin-associated bacteria may constitute opportunistic colonizers. Greater understanding of the amphibian skin microbiome is important for creating effective conservation management programs for animals with declining populations due to skin diseases.

The skin microbiome of amphibians may provide protective effects against skin pathogens [28]. *Batrachochytrium dendrobatidis* is a fungal pathogen that causes chytridiomycosis, which is responsible for amphibian population decline. Although *B*. *dendrobatidis* is associated with altered skin microbiome profiles [129], commensal skin bacteria are known to produce antifungal secondary metabolites that inhibit this pathogen [16]. Members of four bacterial genera (i.e., *Bacillus*, *Chitinophaga*, *Janthinobacterium*, and *Pseudomonas*) were isolated from red-backed salamanders and assayed to determine their ability to prevent *B*. *dendrobatidis* associated clinical signs, as measured by body mass and limb lifting. Whereas all of these bacteria acted synergistically to prevent infection, a co-culture of *Bacillus* and *Chitinophaga* was most effective at inhibiting the fungal pathogen, and this inhibition was linked to production of the metabolite tryptophol [16]. A reduced *P*. *cinereus* cutaneous community on redback salamanders has been linked to clinical signs of disease, namely weight loss and limb lifting [130]. Two closely related frog species (i.e., *Rana sierra* and *Rana muscosa*) were observed to have differential responses to *B*. *dendrobatidis* infections based on distinct skin microbiota. Most *R*. *sierrae* individuals had anti- *B*. *dendrobatidis* bacteria, such as *Pseudomonas*, and were able to persist with *B*. *dendrobatidis* for six years. In contrast, *R*. *muscosa* had lower proportions of this genus and went extinct within a year [131], indicating that *Pseudomonas* may protect frogs from *B*. *dendrobatidis* infections.

Over the past decade, a second distinct species of *Batrachochytrium* was identified as a causative agent for chytridiomycosis [132]. *B*. *salamandrivorans* (Bsal) causes lethal skin disease in salamanders and is responsible for declining populations in Europe [132]. For example, within the Netherlands, only 4% of the fire salamander population remains [133]. The remaining survivors currently do not have an increased resistance to Bsal, which has reservoirs in soil, water, and infected animals [134]. Currently, Bsal has not been detected in North American populations of salamanders [135]. The inability to initially diagnose chytridiomycosis infection in Europe resulted in crucial lost time to implement conservation strategies against the rapidly progressing disease. Researchers of other amphibian populations must therefore consider that chytridiomycosis has multiple known causative agents. Moreover, this finding is broadly applicable to researchers studying skin diseases of humans and other animals.

### Fish

Analyzing the skin microbiota of numerous species of fish can provide insight into the microbial role in host health, which has economic implications for the fishing and aquaculture industries. Early culturing work on North Sea cod (*Gadus morhua*) showed that fish can undergo seasonal variations in skin bacterial abundances [136]. Predominant cultured isolates included *Pseudomonas*, *Achromobacter*, *Corynebacterium*, *Flavobacterium*, and *Vibrio*. The phyla *Proteobacteria*, *Firmicutes*, and *Actinobacteria* dominated several fish species [137]. The core OTU *Aeribacillus* was observed in all fish species, whereas other OTUs reflected species-specific distributions, such as *Microbacterium* in the northern red snapper (*Lutjanus campechanus*) and *Neorickettsia* in the flathead grey mullet (*Mugil cephalus*) [137]. Analysis of wild eel (*Anguilla* spp.) mucus has shown that mucosal pathogens associated with the *Vibrio* genus were highly abundant, implicating wild eels as a niche for their evolution and distribution [138]. A study of 102 fish from six species inhabiting the Gulf of Mexico (*Mugil cephalus*, *Lutjanus campechanus*, *Cynoscion nebulosus*, *Cynoscion arenarius*, *Micropogonias undulatus*, and *Lagodon rhomboides*) determined that each species had a distinct skin community [137].

Seasonal variations of fish skin microbial communities, which at times are coupled with geographic location, have been supported by analyzing lemon sole (*Microstomus kitt*) and skate fish (*Rajidae* spp.) [139] (Fig. 1). These variations may be due to the timing of plankton blooms and changes in the microbial community from the surrounding water. Other factors that may influence the aquatic skin microbiome include pH, dissolved oxygen concentration, and temperature [88, 140]. Fish located in warmer waters have higher proportions of mesophiles [136], whereas those near coast lines possess higher proportions of halotolerant bacteria [140]. Salmon also have varying bacterial loads based on whether they are in marine or freshwater environments [141]. However, a recent study on farmed salmon determined that there was little correlation between the microbial community on the fish and their surrounding water [142]. Manipulating salinity resulted in a reproducible shift in the microbial community that is significantly different from that of surrounding water in the enclosure [143]. As with other vertebrates, geographical location also influenced the bacterial community of six fish species significantly [137].

Fish skin microbiota can also change based on the metabolic status and mutualism of the host (Fig. 1). Salmon (*Salmo salar*) that are deprived of food have significant differences in both bacterial and fungal community composition and density, which was hypothesized to be a result of a decrease in the number of mucosal cells [49]. A study of 44 species of coral reef fish determined that both host diet and phylogeny influenced skin microbial communities [144]. The authors presented two hypotheses to explain this observation. First, various diets may result in shifts in the fish gut microbiome, which would indirectly transfer to the skin in an aquatic environment. Alternately, variations in diet are known to result in changes in the metabolites within surface mucus composition, thereby shifting microbial communities. An additional influence on the fish microbiome is mutualism [145]. Clownfish (*Amphiprioninae* spp.) that associate with anemone undergo a significant shift in their skin microbiota. This reversible shift likely occurs when microorganisms are transferred directly between the animals; however, changes in mucus thickness or chemical substrates may also contribute.

The composition of the fish skin microbiota and related skin pathogens have been studied to prevent large economic losses affecting the fishing and aquaculture industries. For example, colonization of rainbow trout (*Oncorhynchus mykiss*) skin by the pathogen *Vibrio anguillarum* is an important step for disease spread to other body regions [146]. Fish have also been shown to possess beneficial skin bacteria that help prevent infections. For example, rainbow trout have commensal lactic acid bacteria on their skin that prevent *Lactococcus garvieae* from colonizing by producing inhibitory compounds and outcompeting for nutrients [147]. Moreover, the gut health of yellowtail kingfish (*Seriola lalandii*) helps define both skin and gill microbial communities [148]. Fish with chronic lymphocytic enteritis exhibited lower overall diversity and increases in members of the *Proteobacteria* and *Actinobacteria* phyla [148]. Therefore, intestinal diseases have the potential to influence the skin microbiome.

Of methodological concern for fish microbiome studies, fish replicates sampled prior to handling had more variability [142], implying that the process of netting and handling fish alters the sampled skin microbiome. Lack of sex documentation is especially prevalent in fish studies (Additional file 1: Table S1), due to the difficulty of non-invasive sexing methods. Cultivation studies have estimated a range of 10^2^–10^7^ culturable microorganisms/cm^2^ skin [140]. This wide range has been attributed to variations among capture techniques. Trawling leads to larger microbial loads than capture with a baited line, due to sediment contamination from contact with the seabed and release of fish gastrointestinal contents [149]. Additionally, salmon have higher microbial loads in their spawning grounds than their marine habitat due to varying numbers of bacteria in the water [141]. The true skin microbial density of fish is likely several orders of magnitude higher because culturing techniques only enumerate a subset of the total microbial community. These findings should be considered while designing future aquatic microbiome studies.

## Phylosymbiosis and the vertebrate skin microbiome

Phylosymbiosis describes the eco-evolutionary pattern that occurs when the host phylogenetic trees parallel the similarities observed among the corresponding host-associated microbial communities [150]. This pattern can occur via several mechanisms, including maternal transfer, co-speciation, or selection from the external environment via similar diets or behavior [151]. In other words, co-evolution of hosts and host-associated microbial communities is not the only mechanism underlying phylosymbiosis. Several studies of the gut microbiome demonstrate phylosymbiosis across a range of hosts including, hominids [152], mammals [151], birds [153], fish [154], and insects [150]. A key invertebrate gut microbiome study conducted with a controlled laboratory environment demonstrated topological congruence between insect host phylogenies and microbiota dendrograms [150]. However, this type of analysis has more confounding factors for skin microbiome studies, because the skin is constantly exposed to environmental influences. Despite external influences, congruence between host and microbial phylogentic trees and skin microbiota dendrograms, respectively, was recently reported for the Artiodactyla and Perissodactyla mammalian orders [37]. This study provided the first evidence that phylosymbiosis can be detected for vertebrates and their corresponding skin microbial communities.

Subsequent studies have further demonstrated a strong host influence on skin microbiota (Fig. 1). A study of salamanders and frogs in Guatemala and Mexico observed phylosymbiosis at higher host taxonomic levels, such as order, which was not observed within genera and species [155]. Indeed, at lower taxonomic resolution, the habitat geography became the most important influence [155]. In contrast, a study of amphibians from Madagascar observed a greater influence from the ecology (arboreal vs aquatic vs terrestrial) of the frogs compared to phylosymbiosis [156]. Within tropical coral fish, a weak phylosymbiosis pattern was observed [144]. The authors hypothesized that several confounding factors may have contributed to the weak pattern such as the plasticity of the fish immune system, the age of the fish, and health status. Additionally, this study included 138 fish from 44 species. The possibility exists that including more samples per species would strengthen the observed phylosymbiosis patterns. Moreover, conducting phylosymbiosis studies in controlled laboratory environments may be helpful because many environmental confounding factors can be removed. Indeed, a study of three finch species observed that each bird species had distinct skin microbial communities [92], despite identical diets and environmental exposures. Together, such studies contribute to the hypothesis that vertebrates share an evolutionary pattern with their skin microbiome, which is a first step to identifying the mechanisms responsible for phylosymbiosis.

## From contigs to conservation

Skin microbiome research is currently at an exciting crossroads. Researchers now have high-throughput sequencing technologies and standardized protocols to sample and sequence the skin microbiome from a plethora of animals. The next step is to translate such large-scale survey datasets into testable hypotheses with meaningful outcomes that improve the lives of studied animals. One option to advance the field is to follow the processes used in gut microbiome studies, which have progressed from basic surveys of the microorganisms present to studies that manipulate the intestinal environment to establish clinical therapies.

A translatable application for skin microbiome studies involves developing skin probiotics that could protect animals from skin infections. Initial research in skin probiotics determined that certain strains of the human skin commensal bacterium *Propionibacterium acnes* can ferment glycerol to mitigate methicillin-resistant *Staphylococcus aureus* (MRSA) [157]. Currently, endangered amphibian populations have the greatest need for such a product to protect against chytridiomycosis because conservationists do not have the required tools to mitigate population collapses. Several OTUs of interest isolated from amphibian skin are being examined for their ability to prevent Bd infections. *Janthinobacterium*, *Rhodococcus*, and *Pseudomonas* spp. all have the potential to inhibit Bd [158]. A mixed culture with diverse skin commensals may prove critical for conservation efforts. Moreover, exploring the mechanisms behind why Bd and Bsal infect different amphibian hosts may elucidate valuable information on ways to prevent their transmission to susceptible animals. In addition to improving conservation efforts, development of such products would also be beneficial to other animals and could be applicable to the aquaculture industry, animal husbandry, and the pet industry. For example, effectively preventing *Lactococcus garvieae* or *Vibrio anguillarum* infections in aquaculture would be beneficial from an economic perspective as well as providing healthier food for human consumption.

## Conclusions and recommendations for future studies

Although the animal subphylum Vertebrata possesses a highly diverse range of animal species with varying skin physiology, social constructs, and skin conditions, several common trends are apparent. The habitat and geographic location of an animal, maternal effects, and disease status are factors that affect vertebrate skin microbiota. Additionally, biological sex, age, species, and disease state affect a wide range of vertebrates. Recent evidence suggests that phylosymbiosis occurrs between vertebrates and their skin community and is observable within higher host taxonomic classifications such as the order. Studies that sample hosts from evolutionarily distant branches of the vertebrate tree are therefore positioned to analyze phylosymbiosis and the complex interplay of factors that likely contribute to skin microbial community assemblages.

It is crucial to sample skin microbiomes from a wide range of animals to have meaningful baseline data for conservation management programs. Numerous skin diseases have been linked to population declines and threaten the extinction of a variety of animals. More information is needed on the disease transmission mechanisms of both wild and captive animals. Deeper sequencing should therefore be completed on a wider variety of species and include bacteria, archaea, fungi, and viruses. As stated earlier, there are currently very few reptilian skin microbiome studies, despite their numerous skin diseases. Using standardized methodology and rigorous metadata collection, further characterization of skin microbiota for a wide range of vertebrates in both healthy and diseased states will provide crucial baseline data for conservation efforts, with applications extending to animal care in farming and pet industries. Additionally, each of these classes of animals does not exist in a vacuum. Observations and future potential probiotics that work for one species’ skin microbiome may be translatable for conservation of other animals. Researchers are encouraged to delve further than “skin deep” into studies from other animals that may translate to their animal population of interest.

## Additional file

Additional file 1: **Table S1.** Molecular studies investigating the non-human vertebrate skin microbiome. Only studies that used culture-independent methods were included. Studies within a vertebrate clade are listed in alphabetical order according to first author. (DOCX 80 kb)
