# Supplementary material for: The skin microbiome of vertebrates
Source: Microbiome. 2019 May 23;7:79. doi: 10.1186/s40168-019-0694-6 (PMC6533770; doi:10.1186/s40168-019-0694-6)
Supplement: Supplementary file 1 — Table S1. Molecular studies investigating the non-human vertebrate skin microbiome. Only studies that used culture-independent methods were included. Studies within a vertebrate clade are listed in alphabetical order according to first author. (DOCX 80 kb) [file 40168_2019_694_MOESM1_ESM.docx]

Supplemental Table 1: Molecular studies investigating the non-human vertebrate skin microbiome. Only studies that used culture-independent methods were included. Studies within a vertebrate clade are listed in alphabetical order according to first author.

| **Study** | **Species** | **Common names and sample size** | **Body region** | **PCR primers and sequencing platform** | **# sequences obtained (raw unless stated)** | **Dominant taxa** | **Geographic location** | **Biological sex** |
| --- | --- | --- | --- | --- | --- | --- | --- | --- |
| **Mammals** | | | | | | | | |
| (Apprill *et al*., 2011) (1) | *Megaptera novaeangliae* | 19 humpback whales (including 3 health compromised) | Biopsy of dorsal region or sloughed skin from tags/ water skimmings | 27F-B/1492R  16S rRNA gene  ABI 3730XL | Not reported | *Bacteroidetes*  *Tenacibaculum* | North Pacific near Hawaiian islands | 12 males, 4 females, 4 unknown |
| (Apprill *et al*., 2014) (2) | *Megaptera novaeangliae* | 56 humpback whales | Biopsy of upper flank near dorsal fin | 27FB/ 519R 16S rRNA gene  454 pyrosequencing | 754,593 | *Flavobacteria* *Tenacibaculum* *Gammaproteobacteria* *Psychrobacter* | North Atlantic, North Pacific and South Pacific oceans – free-swimming | Not stated- no difference between sex observed |
| (Avena *et al*., 2016) (3) | *Myotis lucifugus, Perimyotis subflavus, Myotis septentrionalis, Myotis sodalis, Myotis volans, Myotis ciliolabrum, Myotis yumanensis, Eptesicus fuscus, Myotis evotis, Lasiurus cinereus, Corynorhinus townsendii* | 70 little brown bats, 5 tricolored bats, 2 northern long-eared bats, 12 Indiana bats,  24 long-legged myotis, 3 western small-footed bats, 23 Yuma myotis, 19 big brown bats, 2 western long-eared bats, 2 hoary bats, 2 Townsend’s big-eared bats | Forearm and muzzle (same swab) | 515F/806R 16S rRNA gene  Illumina HiSeq (eastern US) and MiSeq (Colorado) | Rarefied to 9,800 sequences per sample | *Proteobacteria Bacteroidetes*  *Thermoleophilia* | Virginia, New York, and Colorado, USA | Recorded |
| (Bierlich *et al*., 2017) (4) | *Megaptera novaeangliae* | 89 humpback whales | Upper flank near dorsal region | 515F/806R 16S rRNA gene  Illumina MiSeq | 9,436 to 1,898,477 sequences per sample | *Psychrobacter,*  *Moraxellaceae*  *Tenacibacterium*  *Flavobacterium* | 12 locations within the Western Antarctic Peninsula | Sex was collected |
| (Cardona *et al.,* 2018) (5) | *Lagenhorynchus obliquidens* | 4 Pacific white-sided dolphins | Periumbilicus skin | 515F/806R 16S rRNA gene  Illumina MiSeq | >5000 sequences per sample | *Pasteurellaceae, Peptostreptococcaceae, and Fusobacteriaceae* had fluctuations throughout the day | Chicago, Illinois, USA | 3 females, 1 male |
| (Cheng *et al*., 2015) (6) | *Sarcophilus harrisii* | 23 Tasmanian devils | Chest-abdomen and pouch | 27F/519R 16S rRNA gene  454 pyrosequencing | 1,223,550 | *Firmicutes:*  *Clostridia*  *Bacilli*  *Gamma-Proteobacteria* | Four locations in Tasmania | Number male/female not stated |
| (Chiarello *et al*., 2017) (7) | *Tursiops truncates*  *Orcinus orca* | 4 killer whales, 4 bottlenose dolphins | Dorsal, caudal, and pectoral fins; anal zone | 341F/784R 16S rRNA gene  Illumina MiSeq | 2,198,758 | *Psychrobacter*  *Enhydrobacter*  *Staphylococcus*  *Sphingomonas* | Antibes, France | 2 males and 2 females per specices |
| (Council *et al.*, 2015) (8) | *Pan troglodytes, Gorilla gorilla, Papio, Macaca mulatta* | 7 chimpanzees, 5 gorillas,  11 baboons,  2 rhesus macaques | Axillae | 515F/806R 16S rRNA gene  454 pyrosequencing | 798,818 | *Firmicutes*  *Actinobacteria Proteobacteria*  *Bacteroidetes* | North Carolina zoo, USA. *Rm* = semi-free ranging in Puerto Rico | Unknown |
| (Hoffman *et al.,* 2014) (9) | *Canis lupus familiaris* | 12 healthy and 6 allergic dogs | 12 skin sites (healthy)  4 skin sites (allergic) | 28F/519R 16S rRNA gene  FLX-titanium amplicon pyrosequencing (bTEFAP) | 779,812 | *Proteobacteria* *Oxalobacteriaceae* | Companion animals – Texas, USA | 6 males, 6 females healthy; 4 males, 2 females allergic |
| (Kamus *et al.,* 2018) (10) | *Equus ferus* | 4 mares | Thorax and limb  Wounds had bandaged and unbandaged experimental groups | 564F/785R 16S rRNA gene  llumina MiSeq | 4,321,050  Rarefied to 6,848 per sample | Main genera were unclassified Gp6/ Bacteria, *Planctomyetaceae*, and *Acidobacteria*  Wounds had an increase in *Fusobacteria* and *Actinobacillus* | Montreal, Canada | 4 mares |
| (Klitgaard *et al*., 2008) (11) | *Bos taurus* | 32 beef and dairy cattle | Lower limbs from slaughterhouse | 10FX/1509R 16S rRNA gene  Trep-46F/1509R  ABI 3130 genetic analyzer | Not reported | *Treponema spp.*  *Fusobacterium necrophorum*  *Steptococcus dysgalactiae*  *Pasteurella spp.*  *Klebsiella oxytoca* | Denmark | Unknown |
| (Lavrinienko *et al*., 2018) (12) | *Myodes glareolus* | 157 wild bank voles | Dorsal thorax | 515F/806R16S rRNA gene  Illumina MiSeq | 8,012,677  Rarefied to 10,268 per sample | *Proteobacteria*  *Firmicutes*  *Actinobacteria*  *Bacteroidetes*  *Cyanobacteria* | Ukraine:  Kyiv and Chernobyl Exclusion Zone | 63 males, 94 females |
| (Meason-Smith *et al*., 2017) (13) | *Felis catus* | 11 healthy and 9 allergic cats | 12 skin sites (healthy)  6 skin sites (allergic) | ITS1F/ITS2R  ITS1 region  Illumina MiSeq | 9,770,840 | *Cladosporium*  *Alternaria* | Companion animals – Texas, USA | 5 males, 6 females healthy; 4 males, 5 females allergic |
| (Ross *et al*., 2018) (14) | 38 species | 177 nonhuman mammals from 10 orders | Back, torso, and inner thigh | Pro341F/Pro805R 16S rRNA gene  Illumina MiSeq | 6,550,625  Rarefied to 1,654 per sample | Core OTUs for non human mammals were A*rthrobacter, Sphingomonas,* and *Microbacteriaceae*. Most abundant OTU varied for each species. | Toronto Zoo, African Lion Safari, farms, households, and sourced from the wild in Ontario, Canada | 94 females, 81 males |
| (Russo *et al*., 2018) (15) | *Tursiops truncatus* | 6 free-ranging bottlenose dolphins | Biospies | V1-V3 and V4 region of the 16S rRNA gene  Illumina MiSeq | >3000 reads per sample | *Lachnospiraceae*  *Gammaproteobacteria*  *Pseudomonas*  *Diaphorobacter*  *Acinetobacter*  *Acidovorax*  *Dechloromonas* | Southern California | 4 females, 2 males |
| (Winter *et al*., 2017) (16) | *Myotis ciliolabrum, Myotis*  *californicus, Myotis evotis, Myotis occultus, Myotis thysanodes, Myotis velifer, Myotis volans,*  *Corynorhinus townsendii, Eptesicus fuscus, Tadarida brasiliensis, Antrozous pallidus,*  *Parastrellus hesperus, Lasionycteris noctivagans* | 186 bats from 13 species | Entire skin and furred region including ears, wings, uropatagia | 27F/519R 16S rRNA gene  Roche 454FLX titanium | 843-20,515 sequences per sample | *Actinobacteria*  *Alphaproteobacteria*  *Gammaproteobacteria*  *Firmicutes* | Arizona and New Mexico, USA | 65 female and 95 male |
| (Zeng *et al*., 2017) (17) | *Sus scrofa* | 82 pigs sourced from Tibetan, Rongchang, and Qingyu breeds | Back skin near neck | 515F/806R 16S rRNA gene  Illumina MiSeq | 6,644,855 |  | Daocheng – eastern Tibetan plateau  Sichuan basin, China | Mix of boars and sows |
| (Zinicola *et al*., 2015) (18) | *Bos taurus* | 89 Holstein dairy cows | Punch biopsies of healthy and lesioned hooves | 515F/806R 16S rRNA gene  Illumina MiSeq | 21,345,584 quality filtered sequences | *Firmicutes*  *Spirochaetes*  *Bacteroidetes Actinobacteria Proteobacteria*  *Tenericutes* | Three dairy farms in New York, USA | 89 females |
| **Birds** | | | | | | | | |
| (Denesvre *et al*., 2015) (19) | *Gallus gallus domesticus* | 15 chickens | Side of body protected by feathers | bacteriophage phi29 polymerase using random primers  Illumina HiSeq 2000 | 2.4x10^8^ | Herpesviruses from the *Mardivirus* genus | France – captive | Unknown |
| (Engel *et al*., 2018) (20) | *Taeniopygia gutatta*  *Lonchura striata domestica Stagonopleura gutatta* | 8 zebra finches  11 diamond firetails  12 Bengalese finches | Neck  Preen gland | 341F/785R 16S rRNA gene  Illumina MiSeq | Rarefied to 8,007 sequences per sample | 16 families = 85% reads  Zebra finch = *Methylobacteriaceae*  Diamond & Bengalese finch = *Moraxellaceae* | Bielefield Germany | 16 males, 12 females, 3 unknown |
| (Kulkarni and Heeb, 2007) (21) | *Taeniopygia guttata* | 48 zebra finches | Wing feathers, cloaca, beak | Amplicon sequencing of kerA gene | NA | Only *Bacillus licheniformis* was amplified | Switzerland - caged | 24 males, 24 females |
| (Lucas *et al*., 2005) (22) | *Sturnus vulgaris* | 42 European starlings | Chest feathers were removed | 16S-23S intergenic spacer region  S-D-Bact-1522-b-S-20/L-D-Bact-132- a-A-18 | NA | RISA provides information on bacterial densities | University of Lausanne, Switzerland | 15 males, 27 females |
| (Roggenbuck *et al*., 2014) (23) | *Coragyps atratus, Cathartes aura* | 26 black vultures, 24 turkey vultures | Facial skin | 341F/806R 16S rRNA gene  454-Roche-FLX Titanium | 650,697 trimmed | *Clostridia* and *Fusobacteria* | Nashville, USA | Not stated- no difference between sex |
| **Reptiles** | | | | | | | | |
| (Allender *et al*., 2018) (24) | *Sistrurus catenatus* | 144 Eastern Massasaugas | 36 from lesions  96 from apparently healthy skin from the face | 515F/806R 16S rRNA gene  Fungal ITS2 region  Illumina MiSeq | Rarefied to 9,418 sequences per sample | Alpha diversity for fungi was reduced in samples that were positive for *Ophidiomyces*  *Pseudomonas*  *Burkholderiales, Actinomycetales Enterobacteriaceae* | Carlyle Lake, Illinois | 57 males, 52 females, 1 unknown |
| (Hyde *et al*., 2016) (25) | *Varanus komodoensis* | 38 Komodo dragons | “Designated area of skin” | 515F/806R 16S rRNA gene  Illumina MiSeq | Rarefied to 3,210 sequences per sample | *Proteobacteria*  *Firmicutes*  *Actinobacteria*  *Bacteroidetes*  *Fusobacteria*  *[Thermi]* | Zoos in Atlanta, Bronx, Denver, Fort Worth, Brownsville, Honolulu, Houston, Jacksonville, Los Angeles, Albuquerque, Virginia Beach, and Seattle, USA | Not stated |
| (Stöhr *et al*., 2013) (26) | *Anolis sagrei, Dopasia gracilis, Anolis carolinensis, Iguana iguana, Pogona vitticeps* | 3 brown anoles, 1 Asian glass lizard, 1 green anole, 1 green iguana, 1 central bearded dragon | Skin biopsies from affected regions, mixed organs, small intestine | Ol T1/ Ol T2R of the major capsid protein (MCP) gene  AdVs and IIV were also tested | Presence/absence of viruses reported | *Ranavirus*  *Reovirus*  *Iridovirus*  *Adenovirus* | Germany | Various ratios in case studies |
| **Amphibians** | | | | | | | | |
| (Bataille *et al.,* 2016) (27) | *Bombina orientalis* | 11 wild and 18 captive fire-bellied toads | Ventral area, thigh, toe webbing (1st swab). Back (2^nd^ swab) | ITS1-3 Chytr 5.8S Chytr  GAIIx Illumina sequencer | 1,026,343 | *Proteobacteria*  *Actinobacteria*  *Bacteroidetes* | Gangwon Province, South Korea | Measured |
| (Becker *et al.,* 2014) (28) | *Atelopus zeteki* | 27 wild, 10 captive Panamanian golden frogs | Ventral and dorsal surfaces, thighs, and feet | 515F/806R 16S rRNA gene  Illumina MiSeq | Rarefied to 19,500 sequences per sample | *Proteobacteria*  *Actinobacteria*  *Bacteroidetes* | Wild frogs obtained from Río Mata Ahogado, Panamá; captive from Washingtin,DC,USA | Unknown |
| (Belden *et al.,* 2015) (29) | *Agalychnis callidryas, Dendropsophus ebraccatus, Craugastor fitzingeri, Anaxyrus americanus, Lithobates catesbeianus, Pseudacris crucifer* | 62 red-eyed treefrogs, 53 pantless treefrogs, 21 Fitzinger’s robber frogs, 9 American toads, 35 American bullfrogs, 34 spring peepers | Ventral surface, thigh, hindfoot (one swab) | 515F/806R 16S rRNA gene  Illumina MiSeq | Rarefied to 7000 sequences per sample | *Proteobacteria*  *Actinobacteria*  *Bacteroidetes*  *Firmicutes* | Four sites in Panama and seven site in USA | Unknown |
| (Bletz *et al.,* 2017) (30) | 89 frog species | 800 frogs | Ventral abdomen, ventral thigh, each foot. | 515F/806R 16S rRNA gene  Illumina MiSeq | Rarefied to 4000 sequences per sample | *Proteobacteria*  *Bacteroidetes*  *Actinobacteria*  *Firmicutes* | Madagascar | Unknown |
| (Costa *et al*., 2016) (31) | *Pelophylax perezi* | 31 Perez’s frogs | Ventral and dorsal region, head, lateral region, surface of thigh, foot (1 swab) | 27F/1525R 16S rRNA gene  DGGE | NA | *Actinobacteria*  *Alphaproteobacteria* | Five sites in Portugal | Unknown |
| (Ellison *et al*., 2018) (32) | *Plectrohyla*  *Bolitoglossa*  *Pseudoeurycea* | 23 Spikethumb frogs  19 lungless salamanders  30 false brook salamanders | Entire skin surface | V3-V4 region 16S rRNA gene  Illumina MiSeq | 2,058,236  Rarefied to 7036 sequences per sample | *Flavobacteriales*  *Pseudomonadales*  *Rhizobiales*  *Actinomycetles* | Chiapas, Mexico  Western Guatemala | Unknown |
| (Federici *et al,* 2015) (33) | *Rana italica* | 6 Italian steam frogs: 3 infected, 3 uninfected | Lateral, ventral, and dorsal surfaces of the body, thighs, and feet | 783F/1027R  16S rRNA gene  Illumina HiSeq 1000 | 612,786-1,016,572 reads per sample | *Comamonadaceae Moraxellaceae* *Pseudomonadaceae* | Nestore Valley, Italy | 5 males, 1 female |
| (Fitzpatrick and Allson, 2014) (34) | *Plethodon jordani* | 29 red-cheeked salamanders | Trunk (2 swabs each) | 515F/806R 16S rRNA gene  Hi-Seq 2000 | 1 965 175 after processing | *Gammaproteobacteria*  *Acidobacteria* | North Carolina, USA | Unknown |
| (Hernández-Gómez *et al*., 2016) (35) | *Cryptobranchus alleganiensis bishopi, Cryptobranchus alleganiensis alleganiensis* | Two subspecies of hellbender giant salamander – 6 Ozark, 5 eastern | Dorsal region, plantar surface of foot, wounds | 27F/338R 16S rRNA gene  Illumina HiSeq 2500 | 15,590,201 filtered sequences | *Proteobacteria,*  *Bacteroidetes,*  *Cyanobacteria,*  *Fusobacteria* | Missouri, USA | Unknown |
| (Jani and Briggs, 2014) (36) | *Rana sierrae* | 8-10 per population of Sierra Nevada yellow-legged frogs | Skin (region unspecified) | 8F/338R 16S rRNA gene  Roche/454 GS FLX | 393,119 (experimental) and 159,126 (field) quality filtered sequences | *Actinobacteria Betaproteobacteria*  *Gammaproteobacteria* | Four sites in California, USA | Unknown |
| (Kueneman *et al.*, 2014) (37) | *Anaxyrus boreas, Pseudacris regilla, Taricha torosa,*  *Lithobates catesbeianus, Rana cascadae* | 47 western toads, 30 Pacific tree frogs, 16 California newts, 11 American bullfrogs, 91 cascades frogs | Adults = ventral surface and limbs.  Larvae = entire body | 515F/806R 16S rRNA gene  Illumina HiSeq | 33.4 million | *Bacteroidetes Gammaproteobacteria Alphaproteobacteria Firmicutes*  *Sphingobacteria* *Actinobacteria* | California’s Central Valley and the Trinity Alps in Northern California, USA | Unknown |
| (Lauer *et al*., 2008) (38) | *Hemidactylium scutatum* | 87 four-toed salamanders | Ventral and lateral sides | 357F/907R 16S rRNA gene | Not reported | *Bacteroidetes*  *Firmicutes*  *Actinobacteria*  *Proteobacteria* | Three ponds in Virginia, USA | 87 females |
| (Longo *et al*., 2015) (39) | *Lithobates yavapaiensis, Eleutherodactylus coqui* | 37 lowland leopard frog, 52 common coqui | Standardized number of swab strokes per frog and body region | 16S-23S intergenic spacer region 1406F/ 23S-125R  515F/806R 16S rRNA gene  Illumina MiSeq | 1,179,178 | *Proteobacteria Actinobacteria Bacteroidetes* | Arizona, USA and Puerto Rico | Unknown |
| (Loudon *et al.*, 2014) (40) | *Plethodon cinereus* | 65 red-backed salamanders | Ventral surface | 515F/806R 16S rRNA gene  Illumina HiSeq 2000 | 23.3 million | 5/8 core OTUs were *Pseudomonadaceae* | Virginia, USA | Unknown |
| (McKenzie *et al.,* 2012) (41) | *Lithobates pipiens,*  *Pseudacris triseriata,*  *Ambystoma tigrinum* | 7 northern leopard frogs, 14 western chorus frogs, 12 tiger salamanders (all larval stage) | Entire body | 27F/388R  16S rRNA gene  454 pyrosequencing | Average of 1220 sequences per sample (range 780–1510) | *Acidobacteria Actinobacteria Bacteriodetes Cyanobacteria*  *Firmicutes*  *Proteobacteria* | Four pond habitats in Colorado, USA | Unknown |
| **Fish** | | | | | | | | |
| (Carda-Diéguez *et al*., 2017) (42) | *Anguilla anguilla* | 20 eels from a farm  10,000 glass eels from the wild | Mucus was detached in sterile PBS for 20 min | 616F/699R  454 Roche titanium sequencer and Illumina HiSeq | 3 metagenomes generated | *Actinobacteria*  *Gammaproteobacteria*  *Pseudomonas* maintained similar proportions, while *Vibrio* was abundant in SMS. | Intensive eel farm near Prat de Cabanes-Torreblanca  Wild eels from rivers by Spain’s Atlantic coast | Unknown |
| (Chiarello *et al.,* 2018) (43) | 44 distinct species from 5 orders and 2 families | 138 fish from 44 coral reef species | Back of operculum to caudal peduncle | 515F-806R 16S rRNA gene  Illumina MiSeq | 2450-43,306 sequences per sample | *Gammaproteobacteria*  *was overall the most abundant*  *Microbiome was species specfic* | Coral reefs near Mayotte Island, France in the Indian Ocean. Two sampling sites separated by 15km | Unknown |
| (Larsen *et al.,* 2013) (44) | *Mugil cephalus, Lutjanus campechanus, Cynoscion nebulosus, Cynoscion arenarius, Micropogonias undulatus, Lagodon rhomboides* | 15 striped mullet, 8 red snapper, 11 spotted seatrout, 24 sand seatrout, 27 Atlantic croaker, 17 pinfish | Dorsal fin tissue | RISA  Bact-8F/UNI534R  16S rRNA gene  ABI 3730xl sequencer | Rarefied to 69 sequences per sample | *Proteobacteria*  *Firmicutes*  *Actinobacteria*  *Aeribacillus* observed in all species | Coastal waters of Alabama and Mississippi, USA  Offshore of Louisiana, USA | Unknown |
| (Legrand *et al*., 2018) (45) | *Seriola lalandi* | 36 yellowtail kingfish | Skin along the lateral line and gills | 27F/338R 16S rRNA gene after conversion of RNA to cDNA  Illumina MiSeq | 3,766,157 | *Proteobacteria*  *Bacteroidetes* | Southern Australia commercial aquaculture enterprise | Unknown |
| (Minniti, *et al*., 2017) (46) | *Salmo salar* | 45 salmon | Entire right side of fish | 341F/805R 16S rRNA gene  Illumina MiSeq | 2,690,828 | *Proteobacteria*  *Firmicutes*  *Actinobacteria*  *Lactobacillus*  and *Methylobacterium* were present in almost all samples | Sunndalsøra, Norway | Unknown |
| (Schmidt *et al.,* 2015) (47) | *Poecilia sphenops* | 30 Black Molly fish | Entire fish, including gut | 967F/1064R 16S rRNA gene  Illumina HiSeq | 9.2x10^5^ avg. reads per sample post processing | *Gammaproteobacteria Fusobacteria* | Purchased from supplier in USA | Unknown |

**References**

1. Apprill A, Mooney TA, Lyman E, Stimpert AK, Rappé MS (2011) Humpback whales harbour a combination of specific and variable skin bacteria. *Environ Microbiol Rep* 3(2):223–232.

2. Apprill A, et al. (2014) Humpback whale populations share a core skin bacterial community: towards a health index for marine mammals? *PLoS ONE* 9(3). doi:10.1371/journal.pone.0090785.

3. Avena C V, et al. (2016) Deconstructing the bat skin microbiome: influences of the host and the environment. *Front Microbiol* 7:1753.

4. Bierlich KC, et al. (2017) Temporal and regional variability in the skin microbiome of humpback whales along the western Antarctic peninsula. *Appl Environ Microbiol* 84(5):1–15.

5. Cardona C, et al. (2018) Environmental sources of bacteria differentially influence host-associated microbial dynamics. *mSystems* 3:e00052–18.

6. Cheng Y, et al. (2015) The Tasmanian devil microbiome—implications for conservation and management. *Microbiome* 3(1):76.

7. Chiarello M, Villé S, Bo C, A JC, Bo T (2017) Captive bottlenose dolphins and killer whales harbor a species- specific skin microbiota that varies among individuals. *Sci Rep* 7:15269.

8. Council SE, et al. (2016) Diversity and evolution of the primate skin microbiome. *Proc R Soc B Biol Sci* 283(1822):2586.

9. Hoffmann AR, et al. (2014) The skin microbiome in healthy and allergic dogs. *PLoS ONE* 9(1):e83197.

10. Kamus LJ, Theoret C, Costa MC (2018) Use of next generation sequencing to investigate the microbiota of experimentally induced wounds and the effect of bandaging in horses. *PLoS ONE* 13(11):e0206989.

11. Klitgaard K, Boye M, Capion N, Jensen TK (2008) Evidence of multiple *Treponema* phylotypes involved in bovine digital dermatitis as shown by 16S rRNA gene analysis and fluorescence in situ hybridization. *J Clin Microbiol* 46(9):3012–3020.

12. Lavrinienko A, Tukalenko E, Mappes T, Watts PC (2018) Skin and gut microbiomes of a wild mammal respond to different environmental cues. *Microbiome* 6:209.

13. Meason-Smith C, et al. (2016) Characterization of the cutaneous mycobiota in healthy and allergic cats using next generation sequencing. *Vet Dermatol* 28(June 2016):71-e17.

14. Ross AA, Müller KM, Weese JS, Neufeld JD (2018) Comprehensive skin microbiome analysis reveals the uniqueness of human skin and evidence for phylosymbiosis within the class Mammalia. *Proc Natl Acad Sci USA* 115(25):E5786–E5795.

15. Russo CD, et al. (2018) Bacterial species identified on the skin of bottlenose dolphins off southern California via next generation sequencing techniques. *Microb Ecol* 75:303–309.

16. Winter AS, et al. (2017) Skin and fur bacterial diversity and community structure on American southwestern bats: effects of habitat , geography and bat traits. *PeerJ* 5:e3944.

17. Zeng B, et al. (2017) High-altitude living shapes the skin microbiome in humans and pigs. *Front Microbiol* 8:1929.

18. Zinicola M, et al. (2015) Altered microbiomes in bovine digital dermatitis lesions, and the gut as a pathogen reservoir. *PLoS ONE* 10(3):e0120504.

19. Denesvre C, Dumarest M, Rémy S, Gourichon D, Eloit M (2015) Chicken skin virome analyzed by high-throughput sequencing shows a composition highly different from human skin. *Virus Genes* 51(2):209–216.

20. Engel K, et al. (2018) Individual- and species-specific skin microbiomes in three different estrildid finch species revealed by 16S amplicon sequencing. *Microb Ecol* 76:518–529.

21. Kulkarni S, Heeb P (2007) Social and sexual behaviours aid transmission of bacteria in birds. *Behav Processes* 74(1):88–92.

22. Lucas FS, Moureau B, Jourdie V, Heeb P (2005) Brood size modifications affect plumage bacterial assemblages of European starlings. *Mol Ecol* 14(2):639–646.

23. Roggenbuck M, et al. (2014) The microbiome of new world vultures. *Nat Commun* 5(May):5498.

24. Allender MC, Baker S, Britton M, Kent AD (2018) Snake fungal disease alters skin bacterial and fungal diversity in an endangered rattlesnake. *Sci Rep* 8:12147.

25. Hyde ER, et al. (2016) The oral and skin microbiomes of captive Komodo dragons are significantly shared with their habitat. *mSystems* 1(4):e00046-16.

26. Stöhr AC, et al. (2013) *Ranavirus* infections associated with skin lesions in lizards. *Vet Res* 44(1):84.

27. Bataille A, Lee-Cruz L, Tripathi B, Kim H, Waldman B (2016) Microbiome variation across amphibian skin regions: implications for chytridiomycosis mitigation efforts. *Microb Ecol* 71(1):221–232.

28. Becker MH, Richards-Zawacki CL, Gratwicke B, Belden LK (2014) The effect of captivity on the cutaneous bacterial community of the critically endangered Panamanian golden frog (*Atelopus zeteki*). *Biol Conserv* 176:199–206.

29. Belden LK, et al. (2015) Panamanian frog species host unique skin bacterial communities. *Front Microbiol* 6(OCT):1–21.

30. Bletz MC, et al. (2017) Host ecology rather than host phylogeny drives amphibian skin microbial community structure in the biodiversity hotspot of madagascar. *Front Microbiol* 8:1530.

31. Costa S, Lopes I, Proença DN, Ribeiro R, Morais P V (2016) Diversity of cutaneous bacterial community of *Pelophylax perezi* populations inhabiting different environments. *Sci Total Environ* 572:995–1004.

32. Ellison S, Rovito S, Vredenburg VT (2018) The influence of habitat and phylogeny on the skin microbiome of amphibians in Guatemala and Mexico. *Microb Ecol*:1–11.

33. Federici E, et al. (2015) Characterization of the skin microbiota in Italian stream frogs (*Rana italica*) infected and uninfected by a cutaneous parasitic disease. *Microbes Environ* 30(3):262–269.

34. Fitzpatrick BM, Allison AL (2014) Similarity and differentiation between bacteria associated with skin of salamanders (*Plethodon jordani*) and free-living assemblages. *FEMS Microbiol Ecol* 88(3):482–494.

35. Hernández-Gómez O, Kimble SJA, Briggler JT, Williams RN (2016) Characterization of the cutaneous bacterial communities of two giant salamander subspecies. *Microb Ecol* 73(2):445–454.

36. Jani AJ, Briggs CJ (2014) The pathogen *Batrachochytrium dendrobatidis* disturbs the frog skin microbiome during a natural epidemic and experimental infection. *Proc Natl Acad Sci USA* 111(47):E5049-58.

37. Kueneman JG, et al. (2014) The amphibian skin-associated microbiome across species, space and life history stages. *Mol Ecol* 23(6):1238–1250.

38. Lauer A, Simon MA, Banning JL, Lam BA, Harris RN (2008) Diversity of cutaneous bacteria with antifungal activity isolated from female four-toed salamanders. *ISME J* 2110(10):145–157.

39. Longo A V, Savage AE, Hewson I, Zamudio KR (2015) Seasonal and ontogenetic variation of skin microbial communities and relationships to natural disease dynamics in declining amphibians. *R Soc Open Sci* 2:140377.

40. Loudon AH, et al. (2014) Microbial community dynamics and effect of environmental microbial reservoirs on red-backed salamanders (*Plethodon cinereus*). *ISME J* 8(4):830–40.

41. McKenzie VJ, Bowers RM, Fierer N, Knight R, Lauber CL (2012) Co-habiting amphibian species harbor unique skin bacterial communities in wild populations. *ISME J* 6(3):588–96.

42. Carda-Diéguez M, Ghai R, Rodríguez-valera F, Amaro C (2017) Wild eel microbiome reveals that skin mucus of fish could be a natural niche for aquatic mucosal pathogen evolution. *Microbiome* 6:162.

43. Chiarello M, et al. (2018) Skin microbiome of coral reef fish is highly variable and driven by host phylogeny and diet. *Microbiome* 6:147.

44. Larsen A, Tao Z, Bullard SA, Arias CR (2013) Diversity of the skin microbiota of fishes: evidence for host species specificity. *FEMS Microbiol Ecol* 85(3):483–494.

45. Legrand TPRA, et al. (2018) The inner workings of the outer surface: skin and gill microbiota as indicators of changing gut health in yellowtail kingfish. *Front Microbiol* 8:2664.

46. Minniti G, et al. (2017) The skin-mucus microbial community of farmed atlantic salmon (*Salmo salar*). *Front Microbiol* 8:2043.

47. Schmidt VT, Smith KF, Melvin DW, Amaral-Zettler LA (2015) Community assembly of a euryhaline fish microbiome during salinity acclimation. *Mol Ecol* 24(10):2537–2550.
